# Lateral entorhinal cortex lesions impair odor‐context associative memory in male rats

**DOI:** 10.1002/jnr.25027

**Published:** 2022-02-20

**Authors:** Bjorn M. Persson, Veronika Ambrozova, Stephen Duncan, Emma R. Wood, Akira R. O’Connor, James A. Ainge

**Affiliations:** ^1^ School of Psychology & Neuroscience University of St Andrews St Andrews UK; ^2^ Centre for Discovery Brain Sciences University of Edinburgh Edinburgh UK

**Keywords:** context, episodic memory, hippocampus, odor memory, recognition memory, RRID:AB_2298772, RRID:SCR_013672, RRID:SCR_018321, RRID:SCR_019096

## Abstract

Lateral entorhinal cortex (LEC) has been hypothesized to process nonspatial, item information that is combined with spatial information from medial entorhinal cortex to form episodic memories within the hippocampus. Recent studies, however, have demonstrated that LEC has a role in integrating features of episodic memory prior to the hippocampus. While the precise role of LEC is still unclear, anatomical studies show that LEC is ideally placed to be a hub integrating multisensory information. The current study tests whether the role of LEC in integrating information extends to long‐term multimodal item‐context associations. In Experiment 1, male rats were trained on a context‐dependent odor discrimination task, where two different contexts served as the cue to the correct odor. Rats were pretrained on the task and then received either bilateral excitotoxic LEC or sham lesions. Following surgery, rats were tested on the previously learned odor‐context associations. Control rats showed good memory for the previously learned association but rats with LEC lesions showed significantly impaired performance relative to both their own presurgery performance and to control rats. Experiment 2 went on to test whether impairments in Experiment 1 were the result of LEC lesions impairing either odor or context memory retention alone. Male rats were trained on simple odor and context discrimination tasks that did not require integration of features to solve. Following surgery, both LEC and control rats showed good memory for previously learned odors and contexts. These data show that LEC is critical for long‐term odor‐context associative memory.

## Graphical abstract

Rats with lesions of lateral entorhinal cortex have impaired memory for associations of odors and contexts while their memory for individual items is unaffected. This suggests that integration of associative information occurs at the level of lateral entorhinal cortex as well as the hippocampus.

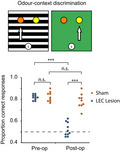

**Significance**

Episodic memory consists of integrated representations of what happened, where we were and the temporal or contextual details of events. Most theories of episodic memory suggest that the integration of these details happens in the hippocampus. Recent evidence has, however, suggested that integration also occurs in lateral entorhinal cortex (LEC). The current findings show that LEC is necessary to remember the associations between odors and contexts. It is not needed to remember odors or contexts by themselves. These findings are of clinical relevance as deficits in episodic memory are one of the first features of Alzheimer’s disease.

## 1 INTRODUCTION

Lateral entorhinal cortex (LEC) is part of the medial temporal lobe memory system. It provides one of the two major inputs to the hippocampus—an area of the brain that has been shown to be critical for episodic and spatial memory (Morris et al., 1982; Vargha‐Khadem et al., 1997). Since the discovery that damage to the hippocampus induces severe and lasting impairments to episodic memory (Scoville & Milner, 1957), a vast literature detailing the role of the hippocampus in memory processing has accumulated (Andersen et al., 2007). Theoretical accounts of information processing within the network have largely focused on how information is funneled into the hippocampus to allow integrated representations of our experience to be created (Davachi, 2006; Diana et al., 2007; Eacott & Norman, 2004; Eichenbaum et al., 2012; Hannula et al., 2013). However, recent studies examining information processing within the rest of the network have challenged the notion that integration of episodic information happens solely in the hippocampus.

Most network models suggest that episodic memory representations within the hippocampus combine spatial information from medial entorhinal cortex (MEC) with nonspatial information from LEC (Eichenbaum et al., 2012; Hasselmo, 2009; Hayman & Jeffery, 2008; Kerr et al., 2007; Knierim et al., 2006). Reports of a variety of clearly spatially modulated signals within MEC (Barry et al., 2007; Hafting et al., 2005; Hoydal et al., 2019; Langston et al., 2010; Sargolini, 2006; Solstad et al., 2008) combined with studies showing a lack of spatial representations within LEC provided support for these models (Hargreaves et al., 2005; Yoganarasimha et al., 2011). However, more recent studies examining LEC have demonstrated a variety of roles for LEC that go beyond processing “nonspatial” information. Increased *c‐fos* expression in LEC has been shown to be correlated with memory of objects within context (Wilson, Langston, et al., 2013). Consistent with this, lesions of LEC have been shown to cause deficits in animals' ability to integrate features of an event including objects, contexts, and spatial locations (Boisselier et al., 2014; Wilson, Langston, et al., 2013; Wilson, Watanabe, et al., 2013), and more specifically fan cells in layer II of LEC have been shown to be critical for integrated object‐place‐context memory (Vandrey et al., 2020). LEC‐lesioned animals also fail to identify changes in complex local environments (Kuruvilla & Ainge, 2017; Rodo et al., 2017; Van Cauter et al., 2012), and show impaired memory in conditioned context aversion learning (Ferry et al., 2015). However, LEC lesions spare the ability to recognize individual “nonspatial” features of events (Kuruvilla & Ainge, 2017; Kuruvilla et al., 2020; Rodo et al., 2017; Wilson, Langston, et al., 2013; Wilson, Watanabe, et al., 2013). These studies suggest that the integration of information needed to form episodic memory is not only a feature of the hippocampus but also happens upstream in LEC.

More recent studies have examined this integration in more detail. LEC has been shown to be critical for using local/proximal but not global/distal features of the environment to form frameworks that will support associative memory (Kuruvilla & Ainge, 2017). This is consistent with reports that neurons within LEC show weak spatial tuning to local environmental features (Neunuebel et al., 2013). LEC has also been shown to be necessary to integrate objects into both 2egocentric and allocentric spatial frameworks with egocentric object‐place memory being especially sensitive to LEC damage (Kuruvilla et al., 2020). A potential mechanism for this deficit is described in studies showing that neurons within LEC respond to the position of objects within an environment (Deshmukh & Knierim, 2011; Tsao et al., 2013) and also to positions in which objects have previously been experienced (Deshmukh & Knierim, 2011). Further studies have also shown evidence for egocentric spatial representations within LEC (Wang et al., 2018).

These studies provide a clear picture where LEC is needed to support memory for integrated representations of objects within a local environment and that could be used to support local egocentric representations. However, it is interesting to view these findings in light of anatomical studies of LEC. Recent reviews of the anatomical literature demonstrate that LEC is one of three cortical hubs that receive extensive cortical inputs consistent with its role in integration of information used to support episodic memory (Bota et al., 2015; Zingg et al., 2014). More detailed examination of these studies shows that some of the major inputs into LEC come from areas processing olfactory information. This raises the question of whether LEC’s involvement in associating features of local spatial frameworks is at least partly due to its role in processing olfactory information, given that olfactory cues are usually more local. Indeed, recent studies have demonstrated that LEC neurons discriminate specific odors even in anesthetized rodents (Leitner et al., 2016).

Here we sought to explicitly test the role of LEC in the integration of olfactory stimuli into local contextual frameworks. A further aim was to extend previous findings, which have largely used spontaneous object exploration tasks. While these are an excellent model of the automatic encoding feature of episodic memory, the data produced are often relatively noisy and the memories tested are relatively short term (Ameen‐Ali et al., 2012; Sivakumaran et al., 2018). The current study examines whether LEC is necessary to remember integrated multisensory features of an event over a longer time period.

## 2 EXPERIMENT 1

### 2.1 Introduction

Experiment 1 sought to test the hypothesis that lesions of LEC cause a deficit in memory for previously learned odor‐context associations. Animals were trained on a biconditional odor‐context discrimination task and the effects of lesions of the LEC were assessed relative to controls on task performance 2 weeks later. Rats were then trained on simple odor and context discrimination tasks to assess whether rats with LEC lesions can learn new simple discriminations.

### 2.2 Methods

#### 2.2.1 Subjects

Twenty‐one male Lister Hooded rats (Harlan & Charles River—average weight at start of experiment = 302 g, approximately 3 months old) were subjects in this experiment (LEC Lesion *n* = 12; sham lesion *n* = 9). The rats were housed in groups of two or three animals per cage, and kept on a 12‐hr light/dark cycle, with testing taking place within the light phase. Animals were kept under food restriction (20 g/day) within 10% of their free feeding weight in order to motivate them to dig for rewards. The study was carried out in compliance with national and international legislation governing the use and maintenance of laboratory animals in scientific research [Animals (Scientific Procedures) Act, 1986; European Communities Council Directive of 2010 (201/63/EU)], under project license 60/4069 and personal license IA4E0C7D4.

#### 2.2.2 Apparatus

**FIGURE 1:**
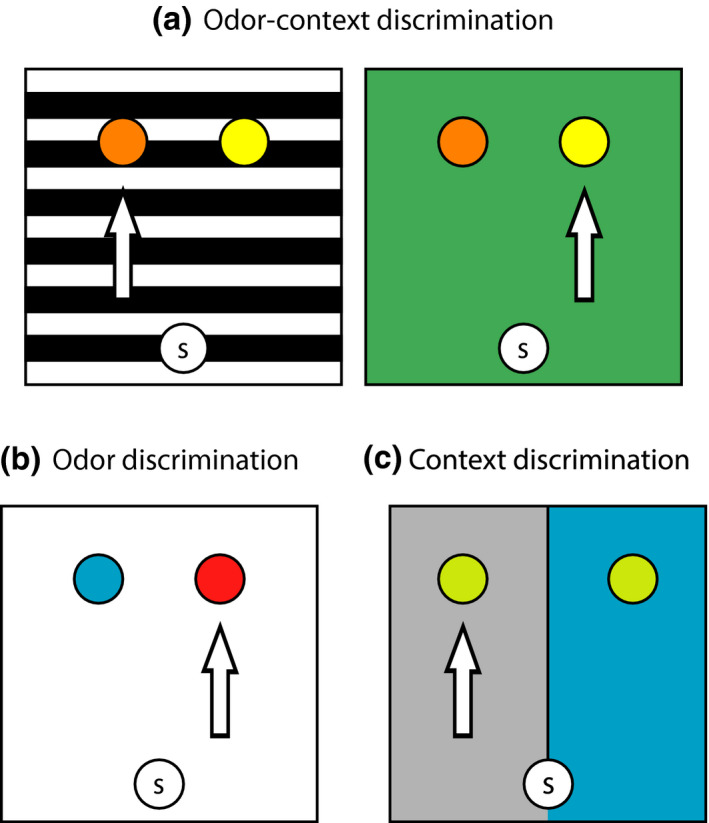
Overview of discrimination tasks. (a) Odor‐context task. The apparatus can be configured with different context cues (walls and floor). Arrows illustrate an example of which odor was rewarded in each context, coriander (orange bowl) or mint (yellow bowl). Odor‐context pairings were counterbalanced across animals. (b) Odor discrimination task. A plain white context was used. In this example, cinnamon (blue) was rewarded and not ginger (red). Correct odor was counterbalanced across animals. (c) Context discrimination task. The box was split into two compartments, that had different context cues, with a divider going down the middle. In this example, the silver context was rewarded. The side of context presentation was counterbalanced across trials and the rewarded context was counterbalanced across animals. The circled S in the panels refers to the rat’s starting position in each trial

Testing took place in a 65 × 65 cm box with 40 cm high interchangeable wall panels giving different sets of contexts. For the odor‐context discrimination task, two sets of contexts were used. The first had white and green checked walls and a black and white striped floor covered with a metal grid. The second context had walls covered in patterned green Christmas wrapping paper and a plain green floor. For the odor discrimination task, a plain white context was used. In the context discrimination task, the box was split into two compartments, one with green sandpaper and one in smooth silver with black dots, divided by a wall going two thirds down the middle. The context was constructed in such way that the spatial locations could be counterbalanced (Figure 1).

Common household spices were mixed with play sand to create odor cues. Spices included: mint (0.9 g/100 ml sand), coriander (0.9 g/100 ml), ginger (0.5 g/100 ml), and cinnamon (0.5 g/100 ml). These odors were selected to give rats distinct odor cues across tasks, avoiding any confusion due to similarity between the odors. Sand was placed in ceramic pots that were fixed to the floor of the apparatus using Dual Lock Velcro (3M™, St. Paul, MN). The pots were 8.5 cm in diameter with a height of 4.3 cm, each pot holding approximately 150 ml of sand.

#### 2.2.3 Habituation

Two pots of unscented sand with buried pieces of chocolate cereal (Weeto, Weetabix, Kettering, UK) were placed in the rats' home cages each morning over 4 days in order to habituate rats to dig for rewards. Rats were then habituated for 10 min per context on 2 days without any pots of sand or rewards present. On the first day of habituation, rats spent 10 min in the checked context, 10 min in the holding cage, and 10 min being handled. This was subsequently repeated with the Christmas context. The second day of habituation followed the same structure, but with the order of the contexts reversed.

#### 2.2.4 Behavioral testing

Across all tasks, a trial consisted of two pots of sand being placed in the box on the far side away from the rat. The rat was placed into the box facing the wall away from the pots of sand (Figure 1). The rat was then allowed to explore the pots of sand until a choice was made, with maximum trial duration of 2 min. If a choice was not made within the 2‐min period, the rat was taken out of the apparatus and the trial was marked as neither correct nor incorrect. A choice was defined as the rat using two paws to dig. Once a choice had been made, the trial was over, and rats were either allowed to eat the reward if their choice was correct, or they were taken out of the box. Between each trial, the sand covering the floor of the box was stirred around and the pots were wiped down with veterinary disinfectant (F10 Products, UK) to cover up any olfactory cues that did not originate from the scented sand in the pots. The correct pot was then rebaited and put into the box. The location of the baited pot was counterbalanced between the left and the right, with the pot not being on either side for more than two trials in a row. Across all tasks, one in 10 trials was a probe trial where no reward was buried in the sand in order to ensure that the animals did not simply dig in the pot where they could smell the reward. Instead, the reward was dropped into the pot if the animal made the correct response.

#### 2.2.5 Presurgery training

##### Odor‐context discrimination

Prior to surgery, rats were trained on the odor‐context discrimination task. Rats were trained to dig for rewards in pots filled with scented sand in two different contexts (Figure 1a). In the first context (checked), a reward could be found in the bowl scented with mint, while no reward was present in the bowl scented with coriander. The opposite rule applied to the second context (Christmas), where digging in coriander gave a reward, and mint gave no reward. Odor‐context pairings were counterbalanced across animals. Training was split into two stages. In the first stage, rats performed up to 60 trials per day, split into six blocks with 10 trials in each block. Contexts remained the same within each block. Animals were allowed to correct their choice during the first four trials in a new context on the first day of training, after this they were taken out of the box following an incorrect choice. When a rat reached a set criterion of eight out of 10 trials correct in one block, the context was changed and the rat was trained until it could reach the same criteria in the other context. If a rat got fewer than eight trials correct, the same context was kept for subsequent blocks until the criterion was reached. As soon as rats could alternate between contexts in at least five out of the six blocks, rats were moved to the second stage where contexts were presented according to pseudo‐randomized schedules for each individual rat, with new schedules being generated for each new day of testing. This criterion was based on the results from a pilot cohort of rats used to develop the procedure, who after meeting the criteria described above demonstrated high levels of accuracy on the pseudo‐randomized context presentation. Rats were trained for 30 trials per day in the pseudo‐random context presentation, with each context presented no more than three times in a row, until they could get 75% of the trials correct on 2 consecutive days. On reaching this criterion, rats underwent surgery.

#### 2.2.6 Postsurgery testing

##### Odor‐Context discrimination

After recovering from surgery, rats were first tested on the previously learned odor‐context associations over 3 consecutive days with the experimenter blind to condition. On each day, rats were given 30 trials of pseudo‐random context presentation, with the same context presented no more than three times in a row.

#### 2.2.7 Odor discrimination

Animals were next trained on a simple odor discrimination task to see whether lesions caused any impairment in odor processing alone. In this task, rats were trained to dig in either cinnamon or ginger in a plain white context to find a reward. Only olfactory information from the sand was required to solve the task and find the reward. Rats were tested for 50 trials per day for 2 days, giving a total of 100 trials. The odor being rewarded was counterbalanced across animals (Figure 1b).

#### 2.2.8 Context discrimination

Rats' ability to discriminate contexts alone following surgery was also assessed. In the context discrimination task, the testing box was split into two compartments—a green and a silver one. Each compartment had a pot filled with odorless sand, and rats were trained to dig for reward in one of the contexts. Rats were trained for 3 consecutive days with 40 trials per day. The sides of the contexts were counterbalanced across trials, with the rewarded context presented on the same side for no more than three consecutive trials (Figure 1c). The identity of the rewarded context was counterbalanced across rat.

#### 2.2.9 Surgery

Rats were initially anesthetized in an induction box using isoflurane (5% isoflurane, 2 L/min O_2_; Abbott Laboratories, Maidenhead, UK) before being placed in a stereotaxic frame (David Kopf Instruments, Tujunga, CA) where anesthesia was maintained via a facemask mounted on the incisor bar (2%–3% isoflurane, 1.2 L/min O_2_). The rat’s head was shaved, an analgesic, Carprieve, was injected subcutaneously (0.05 ml/rat; 5% w/v carprofen, Norbrook Laboratories, UK) before an incision was made along the midline and the skull was exposed. Measurements were taken at both the bregma and lambda to ensure that the skull was level. Holes were drilled bilaterally over the LEC at the following coordinates: AP: −6.5 mm relative to the bregma and ML: ±4.5 mm relative to the midline. The dura was cut using the bent tip of a 30 gauge needle and a glass micropipette (tip diameter 30–40 μm) was inserted at a 10° angle along the ML axis: DV: −6.4 mm relative to the dura. A quantity of 200 nl of ibotenic acid (0.03 M in sterile phosphate buffer, Sigma Aldrich, UK) was injected bilaterally. The pipette was left in situ for 5 min before being retracted. Rats who received sham lesions went through the same procedure as detailed above, but only had the vehicle solution (sterile phosphate buffer) injected in the LEC. Animals were put in a heated box until they recovered from the anesthetic. The following 2 days after surgery an analgesic, Metacam (Boehringer Ingelheim, St Joseph, MO), was mixed into the rats’ food. Animals were left to recover for 10 days before the postsurgery testing began.

#### 2.2.10 Perfusion

Once testing was concluded, rats were deeply anesthetized using (0.9 ml) Pentobarbital (JML, UK) before being perfused transcardially with 50 ml phosphate‐buffered saline followed by at least 250 ml paraformaldehyde (4% made up in 0.1 M phosphate buffer) per rat. After perfusion, brains were removed and placed in 20% sucrose (made up in 0.1 M phosphate buffer) over night.

#### 2.2.11 Histology

Lesions were quantified as previously described (Ainge, Heron‐Maxwell, et al., 2006; Ainge, Keating, et al., 2006). The brains were individually embedded in egg in small tubs and placed in a jar with paraformaldehyde (4%) for approximately 5 days until the egg had fixed to the outside of the brains. Brains were then cut in 50‐μm sections using a freezing microtome. Separate sets of sections were then stained with NeuN (RRID:AB_2298772) and cresyl violet independently, before being mounted on to slides and cover slipped using DPX.

#### 2.2.12 Lesion data analysis

Lesion analysis was made using sections stained with NeuN (RRID:AB_2298772), with cresyl violet‐stained sections used to complement the analysis. Slides were viewed under an Axio Imager 2 light microscope (Carl Zeiss Microscopy), where lesion damaged was defined as a lack of neurons or shrunken cell bodies relative to sham brains. The volume of LEC was calculated by tracing the area of the LEC on 10 sections between −8.28 and −4.68 mm from the bregma in sham‐lesioned animals, using Zen lite imaging software (RRID:SCR_013672). LEC and perirhinal cortex (PRC) were identified using both NeuN (RRID:AB_2298772) and cresyl violet‐stained sections with reference to previously published descriptions of the areas (Burwell et al., 1995) and the Paxinos and Watson (2007) rat brain atlas. The area of the LEC was measured in μm^2^ across both hemispheres, which was then combined to give an estimate of the volume of the LEC throughout the brain. For animals with LEC lesions, the damaged area within the LEC was traced in the same way to get a measure of the extent of the lesions. This measure was then compared to the complete sham volume of the structure to see what percentage of the LEC had been lesioned.

#### 2.2.13 Statistical analysis

Presurgery performance was calculated as the mean accuracy on the last 2 days of training when the animals reached the set criteria. Postsurgery performance was the average accuracy of correct trials over the 3 days of postsurgery testing. Odor‐context memory was analyzed using a mixed factorial ANOVA with group as the between‐subjects factor (LEC and sham lesion) and surgery (pre‐ and post) as the within‐subjects factor. Performance across the 3 days of postsurgery testing on the odor‐context task was also assessed using a mixed factorial ANOVA again with group (LEC and sham lesion) as the between‐subjects factor, and day of testing (1, 2, and 3) as the within‐subjects factor, in order to examine whether the performance of either group improved across time. Performance on unbaited probe trials was compared to baited probe trails using paired samples *t* tests for each group. Odor and context performance was calculated as the average accuracy across the 2 days of odor and 3 days of context testing, respectively. Differences across odor and context discrimination task performance, and across LEC and sham lesion groups were compared using a series of *t* tests.

All variables were assessed for normality using the Shapiro–Wilk test, while equality of variances was tested using Bartlett’s test for normally distributed variables and Levene’s test for nonnormally distributed variables. All analyses met the assumption for normality and equality of variances unless otherwise stated. All statistical analyses were carried out using SPSS Statistics (RRID:SCR_019096). Figures were created using Estimation Stats software (Ho et al., 2019; RRID:SCR_018321).

## 3 RESULTS

### 3.1 Histology results

**FIGURE 2:**
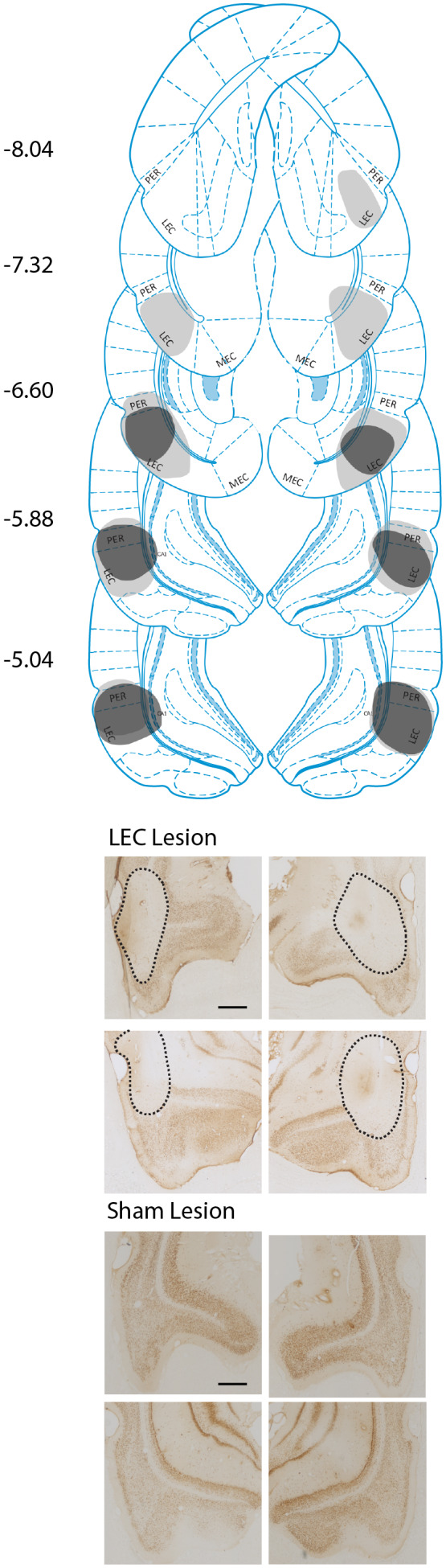
Lesion analysis. (a) Examples of the extent of lesions across the brain. Gray = largest lesion (rat 7) and black = smallest lesion (rat 144). Numbers represent distance from the bregma. (b) Example images showing extent of LEC lesions (top), and the absence of any damage in sham‐lesioned animals (bottom). Representations of coronal sections adapted from Paxinos and Watson (2007). Scale bar in top left image represents 500 μm

From the total sample of 21 rats, 12 LEC lesions and nine sham lesions, histology was inconclusive for four animals due to problems with histological processing: two from the sham lesion group and two from the LEC lesion group. During histology, two additional LEC‐lesioned rats were excluded since lesion damage could not be attributed to the LEC and due to lesions affecting <5% of the LEC and instead showing extensive damage to the PRC and the CA1. This left eight rats with LEC lesions and seven with sham lesions for which full histological analysis could be completed. All eight of the rats in the LEC lesion group had bilateral lesions, ranging from 15.0% to 54.0% of the total volume of the LEC, with the average lesion size being 34.71%. During the histological analysis, it was found that a number of rats had damage to the PRC, as well as the LEC. All rats in the lesion group had some damage to the PRC, ranging from 9.49% to 33.1%, the average lesion size being 20.14% (Table 1). This means that lesion damage was not isolated to the LEC, but instead covered parts of both the LEC and PRC. Some additional damage was seen in the surrounding areas, mainly the CA1; this was, however, estimated to be <5%. None of the rats in the sham lesion group showed any damage to the LEC or surrounding areas (Figure 2). The analyses of behavioral results were first run excluding the four rats for which histology was inconclusive, to examine effects between animals with clear lesion damage or intact brains (LEC; *n* = 8, sham; *n* = 7). All analyses were then rerun including the rats with inconclusive histology (LEC; *n* = 10, sham; *n* = 9). The outcomes across all analyses were the same regardless of whether those four rats were included or excluded, demonstrating that the performance of these animals did not differ from that of the rest of their respective groups. All results reported below include these animals (LEC; *n* = 10, sham; *n* = 9) and the data from these specific animals are highlighted in Figure S1 which demonstrates that these animals' performance was consistent with their respective groups.

**TABLE 1:** Lesion classification and size for the LEC and PRC in Experiment 1

Rat	LEC classification	LEC %	PRC classification	PRC %
1	Bilateral	37.71	Bilateral	22.15
7	Bilateral	54.06	Bilateral	21.61
10	Bilateral	28.79	Bilateral	11.50
21	Bilateral	44.37	Bilateral	23.21
23	Bilateral	33.15	Bilateral	29.77
135	Bilateral	24.77	Unilateral	9.49
140	Bilateral	39.78	Bilateral	33.10
144	Bilateral	15.02	Bilateral	10.28
	Average	34.71	Average	20.14

*Note*: Values indicate average percentage of the area lesioned as compared to the total area of the regions in sham‐lesioned animals.

### 3.2 Behavioral results

#### 3.2.1 Presurgery training

Rats were matched for performance across the two groups to ensure that any differences seen following surgery would not be due to differences in the presurgery training (Table 2). Independent samples *t* tests found no difference between the groups for the number of trials to reach criteria (*t*_(17)_ = −0.50, *p* = 0.63, *d* = 0.24), nor the number of days to criteria (*t*_(17)_ = −0.58, *p* = 0.57, *d* = 0.29). Accuracy between groups' presurgery performance was also compared using an independent samples *t* test, again showing no significant difference (*t*_(17)_ = −0.35, *p* = 0.73, *d* = 0.17).

**TABLE 2:** Mean presurgery performance for LEC and sham lesion groups in Experiment 1

Group	Days to crit.	Trials to crit.	Presurgery
LEC lesion	14.2 (± 3.5)	660 (± 153)	0.818 (± 0.024)
Sham lesion	15.1 (± 3.4)	692 (± 123)	0.824 (± 0.045)

*Note*: Values in brackets are standard deviations.

#### 3.2.2 Postsurgery performance

##### Odor‐context association

**FIGURE 3:**
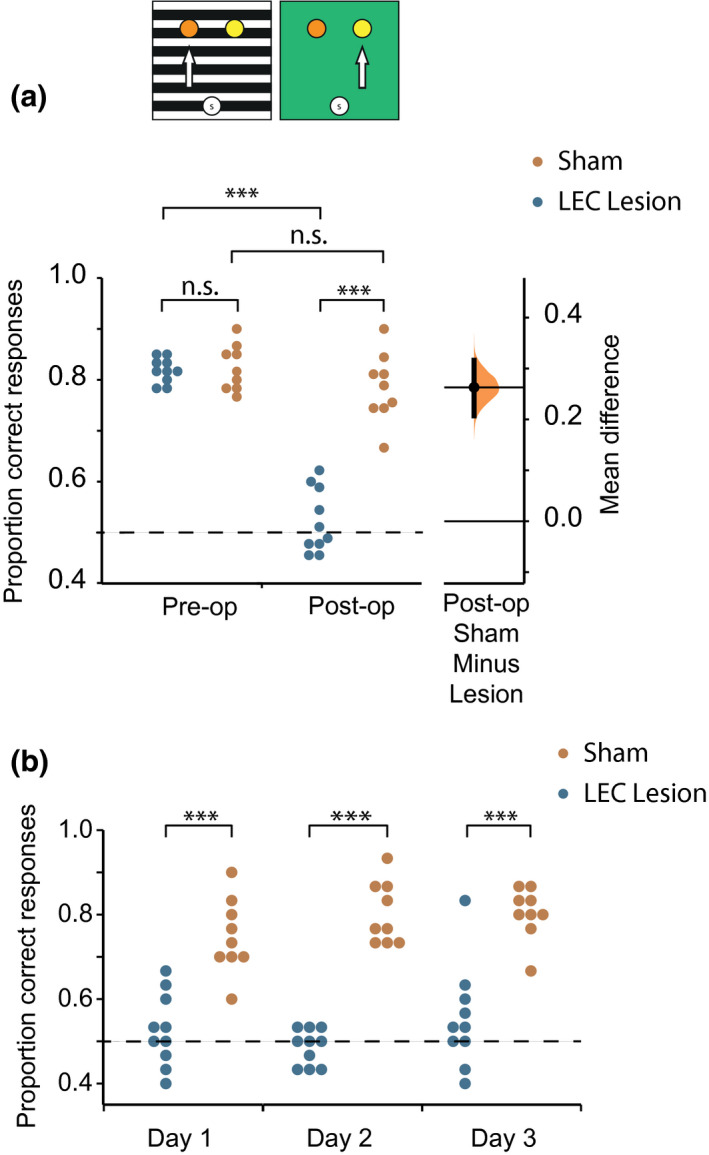
Performance on the odor‐context task pre‐ and postsurgery. (a) Schematic of the task. Mean pre‐ vs. postsurgery performance for the LEC lesion (presurgery: *M* = 0.822, *SD* = 0.024, postsurgery: *M* = 0.535, *SD* = 0.066) and sham lesion (presurgery: *M* = 0.815, *SD* = 0.045, postsurgery: *M* = 0.785, *SD* = 0.067) groups on the odor‐context task. Gardner‐Altman estimation plot depicting effect size as the mean difference between the LEC lesion and sham groups (displayed as a dot) with a 95% confidence interval (indicated by the ends of the vertical error bar) and bootstrap resampling distribution (displayed as a curve). (b) Performance over the 3 days of postsurgery testing on the odor‐context task for the LEC and sham lesion groups. ***p < 0.001

Mean accuracy during the 3 days of postsurgery testing on the odor‐context task dropped in the LEC lesion group, while the accuracy in the sham lesion group remained high (Figure 3a). A 2 (Group: sham vs. Lesion) × 2 (Surgery: pre‐ vs. postsurgery) mixed factorial ANOVA was carried out to examine differences in accuracy. Main effects of Surgery (*F*_(1,17)_ = 155.6, *p* < 0.001, $\eta_{p}^{2}$ = 0.90), and Group (*F*_(1,17)_ = 47.5, *p* < 0.001, $\eta_{p}^{2}$ = 0.74) were found. These main effects were driven by the significant surgery × group interaction (*F*_(1,17)_ = 84.5, *p* < 0.001, $\eta_{p}^{2}$ = 0.83). LEC lesions significantly disrupted memory for odor‐context associations both in comparison to the same rats' performance prior to surgery, as well as rats with sham lesions. Follow‐up pairwise comparisons confirmed this interpretation of the interaction. Paired samples *t* tests showed a significant difference in pre‐ vs. postsurgery accuracy in the LEC lesion group (*t*_(9)_ = 18.1, *p* < 0.001, *d* = 6.71), but not in the sham lesion group (*t*_(8)_ = 1.67, *p* = 0.13, *d* = 0.68). Likewise, an independent samples *t* test found a significant difference in postsurgery accuracy between groups (*t*_(17)_ = −8.83, *p* < 0.001, *d* = 4.14).

The LEC lesion group’s performance was consistently low across the 3 days of postsurgery testing, while sham‐lesioned animals performed consistently better (Figure 3b). A 2 (Group: LEC lesion vs. sham lesion) × 3 (Day: 1,2,3 postsurgery) mixed factorial ANOVA was used to examine whether performance improved over the 3 days of postsurgery testing, either due to remembering or relearning the odor‐context association. There was no significant main effect of Day (*F*_(2,34)_ = 2.30, *p* = 0.12, $\eta_{p}^{2}$ = 0.12), but there was a significant main effect of Group (*F*_(1,17)_ = 78.0, *p* < 0.001, $\eta_{p}^{2}$ = 0.82). The Day × Group interaction was not significant (*F*_(2,34)_ = 2.86, *p* = 0.071, $\eta_{p}^{2}$ = 0.14). Taken together, these effects demonstrate that neither group’s performance changed with time and that animals in the sham lesion group performed significantly better than the LEC lesion group throughout the postsurgery testing.

Performance on nonbaited trials when no reward was placed in the pots showed that both groups performed similarly in these trials as they did in the normal baited trials. Statistical comparison confirmed there were no differences between performance on baited vs. nonbaited trials (*p* < 0.05) for either group. This illustrates that rats did not use the smell of the reward to guide their behavior.

#### 3.2.3 Odor and context discrimination

**FIGURE 4:**
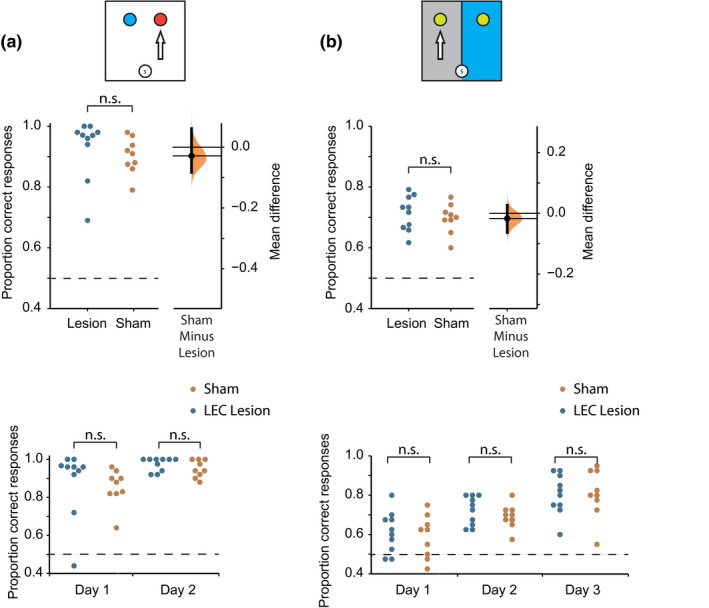
Performance on odor and context discrimination tasks trained postsurgery. (a) Top: Schematic of the odor task. Mean postsurgery performance for the LEC lesion (*M* = 0.931, *SD* = 0.099) and sham lesion (*M* = 0.894, *SD* = 0.057) groups on the odor task. Gardner‐Altman estimation plot depicting effect size as the mean difference between the LEC lesion and sham groups (displayed as a dot) with a 95% confidence interval (indicated by the ends of the vertical error bar) and bootstrap resampling distribution (displayed as a curve). Bottom: Performance over the 2 days of postsurgery training on the odor task for the LEC and sham lesion groups. (b) Top: Schematic of the context task. Mean postsurgery performance for the LEC lesion (*M* = 0.713, *SD* = 0.057) and sham lesion (*M* = 0.697, *SD* = 0.052) groups on the context task. Gardner‐Altman estimation plot depicting effect size as the mean difference between the LEC lesion and sham groups (displayed as a dot) with a 95% confidence interval (indicated by the ends of the vertical error bar) and bootstrap resampling distribution (displayed as a curve). Bottom: Performance over the 3 days of postsurgery training on the context task for the LEC and sham lesion groups

Given the clear deficits in memory for association of odor and context, we next asked whether rats with lesions of LEC were capable of discriminating odors and contexts by themselves. Rats were trained on new odor and context discrimination tasks. Figure 4 shows that both groups of animals performed better on the odor task relative to the context task. It also demonstrates that there was no difference in the accuracy of the two groups learning these new discriminations. This was confirmed with a 2 (Group: sham vs. Lesion) × 2 (Task: Odor vs. Context) mixed factorial ANOVA examining differences in accuracy. There was no significant main effect of Group (*F*_(1,34)_ = 1.01, *p* = 0.32, $\eta_{p}^{2}$ = 0.029) but there was a significant main effect of Task (*F*_(1,34)_ = 88.1, *p* < 0.001, $\eta_{p}^{2}$ = 0.72). The Group × Task interaction was not significant (*F*_(1,34)_ = 0.06, *p* = 0.80, $\eta_{p}^{2}$ = 0.002). It should be noted that the data from the odor task violated the assumption of normality, and as such the results from the analysis of performance on this task should be interpreted with care. These analyses show that while all animals performed better on the odor discrimination than the context discrimination, performance was not affected by lesions of LEC.

Performance of the two groups in both discrimination tasks improved across days of training. For the odor task, a 2 (Group: LEC lesion vs. sham lesion) × 2 (Day: 1,2 postsurgery) mixed factorial ANOVA revealed that there was a significant main effect of day (*F*_(1,19)_ = 10.2, *p* = 0.005, $\eta_{p}^{2}$ = 0.35), no significant main effect of group (*F*_(1,19)_ = 0.89, *p* = 0.36, $\eta_{p}^{2}$ = 0.045), and no significant day × group interaction (*F*_(1,19)_ = 0.084, *p* = 0.78, $\eta_{p}^{2}$ = 0.004). For the context task, a 2 (Group: LEC lesion vs. sham lesion) × 3 (Day: 1,2,3 postsurgery) mixed factorial ANOVA revealed that there was a significant main effect of day (*F*_(2,38)_ = 19.0, *p* = <0.001, $\eta_{p}^{2}$ = 0.500), no significant main effect of group (*F*_(1,12)_ = 0.40, *p* = 0.54, $\eta_{p}^{2}$ = 0.020), and no significant day × group interaction (*F*_(2,38)_ = 0.36, *p* = 0.70, $\eta_{p}^{2}$ = 0.019). Together, this shows that while the rats' performance improved over days in both tasks, there was no difference between groups (Figure 4a,b).

Similar to the odor‐context task, accuracy on probe trials in each task was examined to exclude the possibility that rats used the smell of the reward to select the correct pot. In the odor task, there was no difference in accuracy between baited and nonbaited trials (*p* > 0.05). In the context task, there was also no difference in accuracy between baited and nonbaited trials for the sham group (p > 0.05). However, LEC‐lesioned rats showed significantly lower accuracy in the probe trials relative to the normal baited trials (baited; *M* = 0.71, *SD* = 0.06, unbaited; 0.61, SD 0.10, *t*_(9)_ = 4.05, *p* = 0.003, *d* = 1.25). This was not the case in any of the other tasks for either the LEC or sham groups and so this was not a general problem with the experimental setup. However, this does suggest that LEC‐lesioned rats appeared to use odor cues from the reward to guide behavior in the context task perhaps suggesting impaired context discrimination. However, the lack of group difference in the normal baited context trials and the fact that LEC‐lesioned rats perform significantly above chance in the probe trials (one‐sample *t* test *t*_(9)_ = 3.28, *p* = 0.009, *d* = 1.04) argues against this.

### 3.3 Interim discussion

Experiment 1 demonstrated a clear role for LEC in associative memory for odors and contexts. Animals with lesions of LEC were severely impaired at remembering a previously learned odor‐context association in comparison to control animals who showed good memory. Further experiments show that animals with LEC lesions can learn new discriminations as long as they are not associative in nature. LEC‐lesioned rats were unimpaired in learning simple odor and context discriminations. However, one potential issue is that we tested retention of the odor‐context association while the control studies tested encoding of the simple discriminations. This leaves open the possibility that the deficit seen in LEC‐lesioned rats is better described as a deficit in memory retention for information acquired prior to the lesion, rather than a deficit in associative odor‐context memory. To test this, we carried out a second experiment to examine whether lesions of LEC impair retention for memory of simple odor and context discriminations.

## 4 EXPERIMENT 2

### 4.1 Introduction

Experiment 2 sought to test the hypothesis that lesions of LEC caused a general impairment in memory retention for information acquired prior to the lesion. Rats were trained on simple odor and context discrimination tasks before either LEC or sham lesions were performed. Retention of the nonassociative odor and context stimuli was then tested.

### 4.2 Methods

#### 4.2.1 Subjects

Fourteen male Lister Hooded rats (Charles River, UK, average weight at start of experiment—303 g—approximately 3 months old) were subjects in this experiment (LEC Lesion *n* = 7; sham lesion *n* = 7). The rats were housed in groups of three or four. The rats were kept under the same conditions as in Experiment 1.

Apparatus, habituation, and general testing procedures were as in Experiment 1.

#### 4.2.2 Presurgery training

##### Odor discrimination

Rats were administered 20 trials per day of odor discrimination training until they reached a criterion of 90% correct or better on 2 consecutive days. They were trained to dig in a pot of sand of a particular odor—ginger or cinnamon, counterbalanced across animals.

##### Context discrimination

After they had completed odor discrimination training, rats were administered 20 trials per day of context discrimination training until they reached a criterion of 85% correct or better on 2 consecutive days. They were trained to dig in a pot of odorless sand in a particular context—green or silver, counterbalanced across animals.

#### 4.2.3 Combined training

After rats had completed both odor and context discrimination trainings, they were administered a block of 10 trials on each task per day, until they reached a criterion of 80% correct or better in both tasks on 2 consecutive days. The order in which the tasks were presented was counterbalanced across animals and testing days. The training was resumed 2 days before surgery when the rats were administered 10 trials of each task for 2 days as a reminder.

Surgery, perfusion, histology and lesion analysis, and statistical analysis were carried out as in Experiment 1.

#### 4.2.4 Postsurgery testing

Rats were allowed to recover from surgery for 10 days before testing commenced. They were tested on the odor and context discrimination tasks for 3 days. The order in which the tasks were presented was counterbalanced across animals and testing days. On each day, rats were administered a block of 10 trials on each task.

### 4.3 Results

#### 4.3.1 Histology results

**FIGURE 5:**
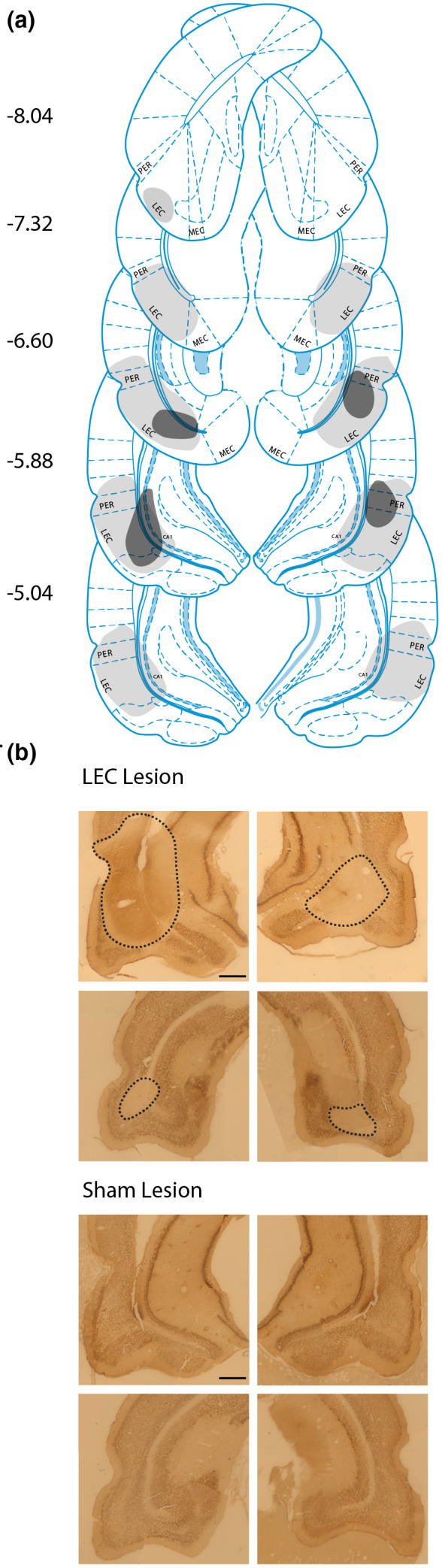
Lesion analysis. (a) Examples of the extent of lesions across the brain. Gray = largest lesion (rat 7), and black = smallest lesion (rat 13). Numbers represent distance from the bregma. (b) Example images showing extent of LEC lesions (top), and the absence of any damage in sham‐lesioned animals (bottom). Representations of coronal sections adapted from Paxinos and Watson (2007). Scale bar in top left image represents 500 μm

The sample consisted of 14 rats, seven lesions, and seven sham lesions. The analysis confirmed that all rats from the experimental group had bilateral lesions. On average, 30.7% of the total LEC volume was lesioned and the extent of lesions ranged from 14.4% to 76.7%. The lesion damage extended to the PRC—on average, 18.9% of the total PRC volume was lesioned and the extent of lesions ranged from 3.2% to 61.3% (Table 3). A set of independent samples *t* tests revealed that the extent of LEC or PRC damage was not significantly different from the extent of LEC damage (*t*_(13)_ = 0.44, *p* = 0.67, *d* = 0.22) or PRC damage (*t*_(13)_ = 0.16, *p* = 0.88, *d* = 0.06), respectively, in Experiment 1. Additionally, there was minor damage to the hippocampal area CA1 but this was estimated to be <5%. None of the animals in the sham lesion group showed any damage to the LEC or surrounding areas (Figure 5).

**TABLE 3:** Lesion classification and size for the LEC and PRC in Experiment 2

Rat	LEC classification	LEC %	PRC classification	PRC %
1	Bilateral	22.06	Bilateral	12.37
5	Bilateral	39.96	Bilateral	6.32
7	Bilateral	76.73	Bilateral	61.27
10	Bilateral	14.52	Bilateral	12.10
13	Bilateral	14.35	Unilateral	3.25
15	Bilateral	15.69	Bilateral	9.35
16	Bilateral	31.49	Unilateral	27.60
	Average	30.69	Average	18.89

*Note*: Values indicate average percentage of the area lesioned as compared to the total area of the regions in sham‐lesioned animals.

#### 4.3.2 Behavioral results

##### Presurgery training

Following presurgery training, rats were matched for performance across the two groups before experimental manipulation (Tables 4 and 5). A set of independent samples *t* tests showed that there was no difference between the groups in the number of trials to reach criterion in the odor discrimination task (*t*_(12)_ = 1.58, *p* = 0.14, *d* = 0.85) or in the context discrimination task (*t*_(12)_ = 0.071; *p* = 0.95; *d* = 0.038); there was no difference between the groups in the number of days to reach criteria criterion in the odor discrimination task (*t*_(12)_ = 1.00; *p* = 0.34, *d* = 0.54) or in the context discrimination task (*t*_(12)_ = 0.35; *p* = 0.74, *d* = 0.19); and there was no difference between the groups in the accuracy of presurgery performance in the odor discrimination task (*t*_(12)_ = 0.34, *p* = 0.74, *d* = 0.18) or in the context discrimination task (*t*_(12)_ = 0.18, *p* = 0.86, *d* = 0.096).

**TABLE 4:** Average presurgery performance of sham and lesion groups on odor discrimination task in Experiment 2

Group	Days to crit.	Trials to crit.	Presurgery
LEC lesion	5.4 (± 0.5)	68 (± 11)	0.950 (± 0.058)
Sham lesion	6.0 (± 1.4)	80 (± 123)	0.941 (± 0.034)

*Note*: Values in brackets are standard deviations.

**TABLE 5:** Average presurgery performance of sham and lesion groups on context discrimination task in Experiment 2

Group	Days to crit.	Trials to crit.	Presurgery
LEC lesion	9.6 (± 2.4)	179 (± 54)	0.935 (± 0.071)
Sham lesion	9.1 (± 2.2)	181 (± 43)	0.941 (± 0.046)

*Note*: Values in brackets are standard deviations.

#### 4.3.3 Postsurgery performance

##### Odor discrimination task

**FIGURE 6:**
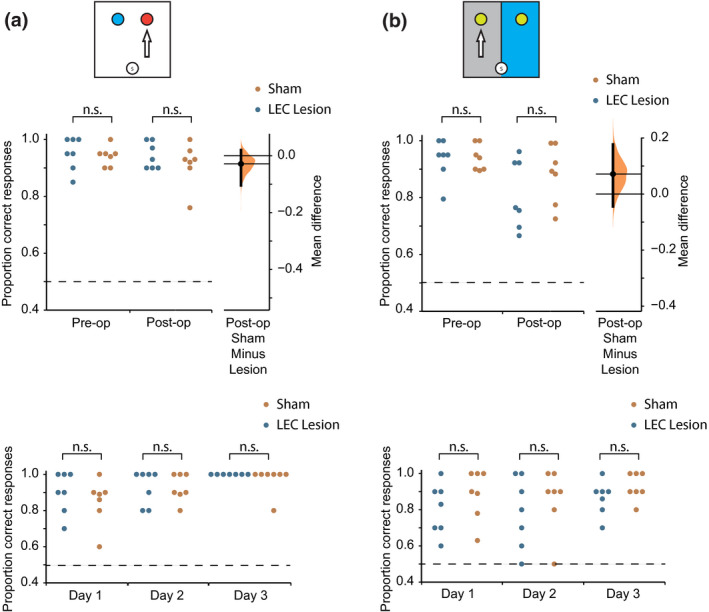
Performance on odor and context discrimination tasks trained presurgery. (a) Top: Schematic of the odor task. Mean pre vs. postsurgery performance for the LEC lesion (presurgery: *M* = 0.950, *SD* = 0.058, postsurgery: *M* = 0.943, *SD* = 0.046) and sham lesion (presurgery: *M* = 0.941, *SD* = 0.034, postsurgery: *M* = 0.914, *SD* = 0.075) groups on the odor task. Gardner‐Altman estimation plot depicting effect size as the mean difference between the LEC lesion and sham groups (displayed as a dot) with a 95% confidence interval (indicated by the ends of the vertical error bar) and bootstrap resampling distribution (displayed as a curve). Bottom: Performance over the 3 days of postsurgery training on the context task for the LEC and sham lesion groups. (b) Top: Schematic of the context task. Mean pre vs. postsurgery performance for the LEC lesion (presurgery: *M* = 0.936, *SD* = 0.069, postsurgery: *M* = 0.819, *SD* = 0.122) and sham lesion (presurgery: *M* = 0.941, *SD* = 0.045, postsurgery: *M* = 0.890, *SD* = 0.103) groups on the context task. Right: Gardner‐Altman estimation plot depicting effect size as the mean difference between the LEC lesion and sham groups (displayed as a dot) with a 95% confidence interval (indicated by the ends of the vertical error bar) and bootstrap resampling distribution (displayed as a curve). Bottom: Performance over the 3 days of postsurgery training on the context task for the LEC and sham lesion groups

Both groups of animals showed good memory for the previously learned odor discrimination (Figure 6a). A 2 (Group: sham vs. Lesion) × 2 (Surgery: pre‐ vs. postsurgery) mixed factorial ANOVA showed no significant main effect of surgery (*F*_(1,12)_ = 0.69, *p* = 0.42, $\eta_{p}^{2}$ = 0.055), no significant main effect of group (*F*_(1,12)_ = 0.76, *p* = 0.40, $\eta_{p}^{2}$ = 0.059), and no significant surgery × group interaction (*F*_(1,12)_ = 0.24, *p* = 0.64, $\eta_{p}^{2}$ = 0.019). This shows that the accuracy of lesion and sham groups in odor discrimination task was comparable before and after surgery (Figure 6a).

Performance on the odor task did improve over days but there was no difference between groups. This was confirmed by a 2 (Group: LEC lesion vs. sham lesion) × 3 (Day: 1,2,3 postsurgery) mixed factorial ANOVA, which revealed that there was a significant main effect of day (*F*_(2,24)_ = 6.47, *p* = 0.006, $\eta_{p}^{2}$ = 0.35), no significant main effect of group (*F*_(1,12)_ = 0.65, *p* = 0.44, $\eta_{p}^{2}$ = 0.051), and no significant day × group interaction (*F*_(2,24)_ = 0.31, *p* = 0.73, $\eta_{p}^{2}$ = 0.025). Together, this shows that while the rats' performance improved over days, there was no difference between groups (Figure 6a). Performance on nonbaited trials when no reward was placed in the pots showed that both groups performed similarly in these trials as they did in the normal baited trials. Statistical comparison confirmed there were no differences between performance on baited vs. nonbaited trials (*p* < 0.05) for either group. This illustrates that rats did not use the smell of the reward to guide their behavior.

##### Context discrimination task

Performance in the context task showed a similar pattern of results to the odor task with no differences in accuracy between groups (Figure 6b). A 2 (Group: sham vs. Lesion) × 2 (Surgery: pre‐ vs. postsurgery) mixed factorial ANOVA showed that there was a significant main effect of surgery (*F*_(1,12)_ = 5.21, *p* = 0.041, $\eta_{p}^{2}$ = 0.30), no significant main effect of group (*F*_(1,12)_ = 1.57, *p* = 0.23, $\eta_{p}^{2}$ = 0.12), and no significant surgery × group interaction (*F*_(1,12)_ = 0.79, *p* = 0.39, $\eta_{p}^{2}$ = 0.062). In contrast to the odor task, the significant main effect of surgery shows that the performance of both groups decreased following surgery. However, there was no difference between sham and LEC lesion groups demonstrating that this change in performance postsurgery is not a result of damage to LEC. This likely reflects increased difficulty of the context task relative to the odor task which manifests in higher rates of forgetting following the retention period. This is in line with the presurgery performance which showed that on average it took the animals fewer days to learn the odor task (*M* = 5.7, *SD* = 0.5) than the context task (*M* = 9.4, *SD* = 2.2). However, while there was a significant decrease by both groups postsurgery, it should be noted that they were still performing significantly above chance‐level performance, demonstrating that the context discrimination had not been completely forgotten (*p* < 0.001 for both groups).

To examine postsurgery performance in the context discrimination in more detail, a 2 (Group: LEC lesion vs. sham lesion) × 3 (Day: 1,2,3 postsurgery) mixed factorial ANOVA was performed. There was no significant main effect of day (*F*_(2,24)_ = 1.92, *p* = 0.17, $\eta_{p}^{2}$ = 0.14), no significant main effect of group (*F*_(1,12)_ = 1.39, *p* = 0.26, $\eta_{p}^{2}$ = 0.10), and no significant day × group interaction (*F*_(2,24)_ = 0.028, *p* = 0.97, $\eta_{p}^{2}$ = 0.002). This shows that both groups did not improve during the postsurgery testing which suggests that the context task is more difficult to relearn/remember compared to the odor task (Figure 6b). Finally, it was investigated whether postsurgery performance in the context discrimination task could have been affected by the presence/absence of odor cues of a reward. There were no differences between performance on baited versus nonbaited trials (*p* < 0.05) for either group.

###### DISCUSSION

Most models of episodic memory processing suggest that information needed to form episodic memory is integrated within the hippocampus (Davachi, 2006; Diana et al., 2007; Eichenbaum et al., 2012). These models take various forms but usually consist of spatial/contextual information from MEC being combined with nonspatial, item information from LEC in the hippocampus (Diana et al., 2007; Eichenbaum et al., 2012). However, recent studies have suggested that LEC is also necessary for this integration (Boisselier et al., 2014; Wilson, Langston, et al., 2013; Wilson, Watanabe, et al., 2013). The present study draws on recent findings, including anatomical studies, which suggest that LEC is ideally placed to be a hub for integrating local multisensory cues into a local spatial framework. We use a multisensory item‐in‐context memory task which has added benefit of producing a long‐lasting memory in contrast to the short‐term memory studies using object exploration studies which are standard in the field.

Rats with lesions of LEC were severely impaired on the odor‐context discrimination task relative to control rats. Their ability to discriminate new odors and contexts, however, was not impaired, demonstrating that the deficit seen in the odor‐context recognition task was not due impairment in discriminating individual features of event. These results are consistent with previous data suggesting that integration of episodic information is not confined to the hippocampus but also happens at the level of LEC (Kuruvilla & Ainge, 2017; Rodo et al., 2017; Van Cauter et al., 2012; Wilson, Langston, et al., 2013; Wilson, Watanabe, et al., 2013). Single neuron recording studies within LEC provide a potential mechanism for this integration by demonstrating that multiple features of events including odors, locations, contexts, and time are integrated at the level of a single neuron (Deshmukh & Knierim, 2011; Deshmukh et al., 2012; Keene et al., 2016; Tsao et al., 2013, 2018). Anatomical studies show that LEC is ideally placed to be a hub for multisensory integration (Canto et al., 2008; Van Strien et al., 2009) and the current studies extend previous findings to show that this integration includes binding olfactory information to local contextual cues. This is also consistent with studies showing that LEC is necessary for integration of odor and tactile stimuli (Boisselier et al., 2014). The current study reinforces previous studies that demonstrate that LEC is not needed for encoding single features of an event. Wilson, Langston, et al. (2013) and Wilson, Watanabe, et al. (2013) showed that LEC‐lesioned rats can remember individual objects and locations, while some studies suggest that LEC lesions can even facilitate odor discrimination by extending the mnemonic trace over longer delays (Ferry et al., 1996, 2006; Wirth et al., 1998), and the current data extend these findings to show that LEC is also not needed to remember odors or contexts. These data support the suggested role for LEC in integration of these features.

Much of the previous data examining the role of LEC in associative recognition are from experiments that used variants of the object recognition task. These spontaneous recognition tasks are ideal for modeling the automatic encoding properties of episodic memory in humans (Morris & Frey, 1997; Sivakumaran et al., 2018) but usually only test memory over short retention intervals of under 1 hr. The current study examined a more robust memory that was shown to be intact in control animals for over 2 weeks. Deficits in LEC‐lesioned rats in this longer lasting memory implicate LEC in long‐term memory for integrated features of our experience.

When interpreting the current findings, it is important to note that all animals within the LEC group had damage to PRC and as such it is important to examine the potential role of PRC in recognition memory. PRC has been consistently shown to be necessary for object recognition (Barker et al., 2007; Buckley & Gaffan, 1997, 1998; Kesner et al., 2001; Norman & Eacott, 2005) and has also been shown to be necessary for remembering contextual cues (Bachevalier et al., 2015; Bucci et al., 2000, 2002; Burwell et al., 2004; Corodimas & LeDoux, 1995; Lee & Lee, 2013). The fact that the animals in the current study were unimpaired at discriminating items and contexts individually suggests that PRC damage in the current experiment is not sufficient to impair either contextual or olfactory learning or memory. The evidence examining the role of PRC in associative recognition memory is more mixed. While damage to PRC has been shown to impair item‐place memory (Barker & Warburton, 2008; Bussey et al., 2001; Lee & Park, 2013) and item‐context memory (Heimer‐McGinn et al., 2017), other studies have found that PRC is not needed to recognize associations of objects with the places and nonspatial contexts in which they are experienced (Eacott & Norman, 2004; Norman & Eacott, 2005). Similarly, while some studies have reported a role for PRC in discrimination of odors (Herzog & Otto, 1997, 1998) others have reported no effect of PRC lesions on odor discrimination (Albasser et al., 2011). One interesting possibility that has been previously suggested is that PRC and the adjacent PRC interact to produce contextual representations (Burwell et al., 2004). Support for this suggestion comes from studies showing that PRC is necessary to remember complex multifeature stimuli that act as contexts in fear conditioning studies (Kholodar‐Smith et al., 2008; Lindquist et al., 2004). It is possible that these contextual representations are bound with items, spatial locations, and even time in LEC. This leaves a level of uncertainty as to the exact role of the PRC in associating features of episodic memory. Given the consistent damage to PRC in the current study, we cannot rule out the possibility that the deficits in odor‐context recognition that we report are at least partially due to disrupted PRC function. Further research will be needed to examine the precise roles of LEC and PRC in associative memory and how these structures interact to produce integrated representations of our experience. What is clear is that models of information processing within this network that suggest that integration occurs exclusively within the hippocampus need reconsideration.

This conclusion is consistent with the literature examining the role of the hippocampus in configural learning. Configural association theory originally suggested that the role of the hippocampus was to combine elementary stimulus events to create unique configural representations that differ from the simple associative strength between elements (Sutherland & Rudy, 1989). However, several later studies showed that animals with lesions of the hippocampus could form configural representations (Alvarado & Rudy, 1995; Gallagher & Holland, 1992; McDonald et al., 1997; Whishaw & Tomie, 1991). This led to the suggestion that the hippocampus and cortex coordinate to process configural representations and that the critical site for these associations is outside of the hippocampus (O'Reilly & Rudy, 2001; Rudy & Sutherland, 1995). The current data suggest that this critical site might be LEC. Another interesting point relating to elemental vs. configural representations is the nature of context within episodic memory. Future studies should aim to examine whether context has a special property that allows the disambiguation of episodic memories or if it is better viewed mechanistically as a configural representation of unique combinations of elements.

However, despite evidence showing that the hippocampus is not needed for configural learning, studies have shown it to be critically important for the integration of episodic information including items, spatial locations, and contexts (King et al., 2002; Langston & Wood, 2010; Mishkin et al., 1998; Mumby et al., 2002; Piterkin et al., 2008). Of particular interest in light of the current findings are studies showing that ventral hippocampus has an important role in associating odors and contexts (Aqrabawi & Kim, 2018; Komorowski et al., 2013; Levinson et al., 2020). This suggests that interactions between ventral hippocampus and LEC may be important to integrate this episodic information. Some rats in the current study did have minor damage to ventral CA1 but their pattern of behavior was similar to the animals with only LEC damage suggesting that the deficit reported here was not driven by damage to the ventral hippocampus. A recent study from Igarashi et al. (2014) demonstrated that coherence of activity between LEC and the hippocampus evolves as rats learn to associate odors with spatial locations. Network coherence is linked to task performance and network representations of unique trial outcomes. Clearly, communication between LEC and hippocampus is critically important for the integration of features of episodic memory. Again, this suggests that we may need to reconsider models of MTL function where integration of the features of episodic memory happens exclusively in the hippocampus.

One final issue to address is that of complexity. Both LEC and PRC have been shown to be important when processing complex stimuli with difficult and more complex discriminations being impaired by damage to these areas (Bartko et al., 2007a, 2007b; Feinberg et al., 2012; Rodo et al., 2017). In the current study, the odor and context discriminations would appear to be less complex than the odor‐context discrimination and so deficits in the associative task could be due to increased complexity. However, the accuracy scores for the context task were lower than those for either the odor‐context or the odor task which would argue against this interpretation. Future studies could aim to dissociate the association and complexity theories by designing complex tasks that do not require the features to be integrated.

The current study presents compelling evidence that LEC is critical for retention of odor‐context associations. It also shows that LEC is not needed for either the encoding or retrieval of single items (odors or contexts). These data are consistent with other studies showing a role of LEC in encoding associations of features of events, while leaving memory for single items unaffected (Wilson, Langston, et al., 2013; Wilson, Watanabe, et al., 2013). This view is consistent with a review and meta‐analysis of LEC function that concluded it is needed for both encoding and retrieval of associations (Morrissey & Takehara‐Nishiuchi, 2014).

###### DECLARATION OF TRANSPARENCY

The authors, reviewers and editors affirm that in accordance to the policies set by the *Journal of Neuroscience Research*, this manuscript presents an accurate and transparent account of the study being reported and that all critical details describing the methods and results are present.

## CONFLICT OF INTEREST

The authors have no conflict of interests.

## AUTHOR CONTRIBUTIONS

All authors had full access to all the data in the study and take responsibility for the integrity of the data and the accuracy of the data analysis. *Conceptualization*, B.P., V.A., and J.A.; *Methodology*, B.P., V.A., and J.A.; *Investigation*, B.P., V.A., S.D., and J.A.; *Formal Analysis*, B.P. and V.A.; *Resources*, J.A.; *Data Curation*, V.A.; *Writing – Original Draft*, B.P., V.A., E.W., A.O., and J.A.; *Writing – Review & Editing*, B.P., V.A., E.W., A.O., and J.A.; *Visualization*, B.P., V.A., and J.A.; *Supervision*, E.W., A.O., and J.A.; *Funding Acquisition*, B.P., V.A., E.W., A.O., and J.A.

### PEER REVIEW

The peer review history for this article is available at https://publons.com/publon/10.1002/jnr.25027.

## Supporting information


**FIGURE S1** Performance on the odour‐context task pre‐ and post‐surgery; performance of animals whose histology could not be quantified is depicted in red. Top: Schematic of the task. Bottom: Mean pre‐ vs. post‐surgery performance for the LEC lesion (pre‐surgery: *M* = 0.822, *SD* = 0.024, post‐surgery: *M* = 0.535, *SD* = 0.066) and sham lesion (pre‐surgery: *M* = 0.815, *SD* = 0.045, post‐surgery: *M* = 0.785, *SD* = 0.067) groups on the odour‐context task. Gardner‐Altman estimation plot depicting effect size as the mean difference between the LEC lesion and sham groups (displayed as a dot) with a 95% confidence interval (indicated by the ends of the vertical error bar) and bootstrap resampling distribution (displayed as a curve)

**TABLE S1** Key resources

Transparent Science Questionnaire for Authors

## Data Availability

The data that support the findings of this study are openly available in University of St Andrews Research Portal at https://doi.org/10.17630/8d6d7a80‐25f4‐4f1e‐8a96‐c433a4916c8b.
