# Supplementary material for: Lateral entorhinal cortex lesions impair odor‐context associative memory in male rats
Source: J Neurosci Res. 2022 Feb 20;100(4):1030–46. doi: 10.1002/jnr.25027 (PMC9302644; doi:10.1002/jnr.25027)
Supplement: Supplementary file 2 — TABLE S1 Key resources [file JNR-100-1030-s001.docx]

| Resource | Source | Identifiers | Additional information |
| --- | --- | --- | --- |
| Anti-Neun, clone A60 | Merck | Cat # MAB377,  Lot #s 2428671; 3278580 RRID: AB_2298772 | Raised in mouse; IgG_1_ isotope; molecular weight: 46-48kDA; positive control: brain tissue; negative control: non-neuronal tissue – e.g. fibroblasts |
| ZEN | Zeiss | RRID: SCR_013672 |  |
| SPSS Statistics | IBM | RRID: SCR_019096 |  |
| Estimation Stats | www.estimationstats.com | RRID: SCR_018321 |  |

Supplementary Table 1: Key resources
